# Supplementary figures and images for: Dynamic organization of Herpesvirus glycoproteins on the viral envelope revealed by super-resolution microscopy
Source: PLoS Pathog. 2019 Dec 2;15(12):e1008209. doi: 10.1371/journal.ppat.1008209 (PMC6907858; doi:10.1371/journal.ppat.1008209)

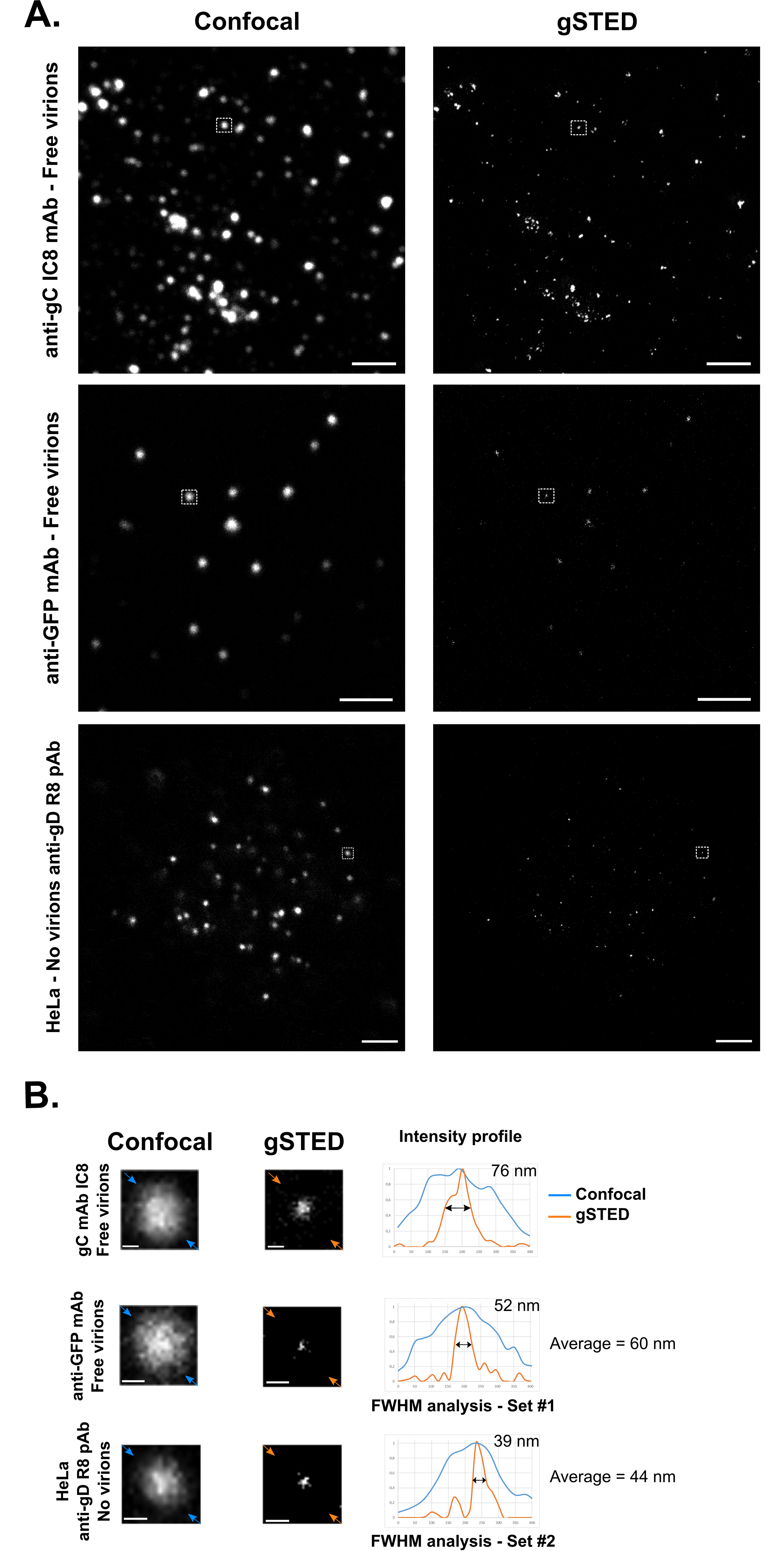

Supplement: S1 Fig — (A) Free virions were attached to glass coverslips and incubated with mAb IC8 against gC or irrelevant anti-GFP monoclonal antibodies. In addition, uninfected HeLa cells were incubated with pAb R8 against gD. All samples were incubated with Oregon-green 488-conjugated secondary antibodies. The nonspecific signal consisting essentially of immune complexes was then imaged using the diffraction limited confocal mode, or the gSTED set-up using the same conditions as those described for imaging of glycoproteins. Scale bar: 2 μm. (B) Enlargement of the regions boxed in A and the corresponding intensity profiles shown along a line of 400 nm. Scale bars: 200 nm. To determine the resolution of the gSTED set-up, the full-width at half maximum (FWHM) was calculated for six different images per set of experiments. One is illustrated here for each set. The average of FWHM was 60 nm for the first set of experiments (52 nm shown here) and 44 nm for the second set of experiments (39 nm here). (TIF) [file ppat.1008209.s001.tif]

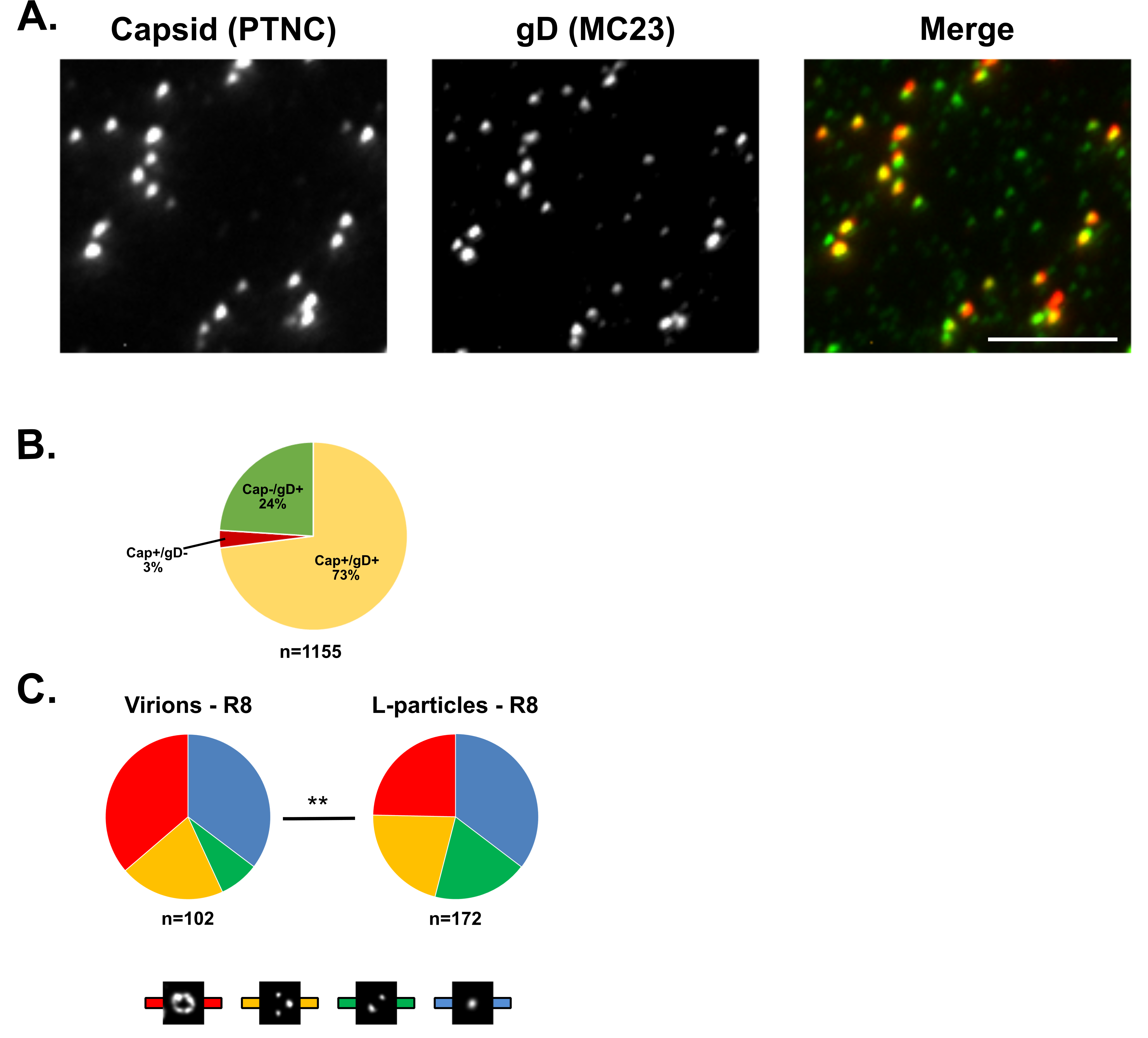

Supplement: S2 Fig — (A) Preparations of purified virus particles were attached to glass coverslips at room-temperature, fixed, permeabilised and labeled with antibody MC23 against gD (green) and antibody PTNC against capsids (red). Scale bars: 5 μm. (B) Quantification of the percentage of virions, L-particles and capsids in 17+ virion preparations. Virions were defined as particles positive for both capsid (PTNC) and gD (MC23) signals (yellow), L-particles (green) were defined as negative for capsid and positive for gD and isolated capsids (red) were defined as positive for capsid and negative for gD. (C) 17+ viral particles were banded on a Ficoll gradient to separate virions from L-particles. Particles were attached to glass coverslips at room-temperature and labeled with anti-gD polyclonal antibody R8. The distribution of gD according to the pattern defined in Fig 2 is shown. A Pearson’s chi-squared test was used to determine whether the profile of distribution between virions and L-particles was significantly different. The p-value indicates the likelihood of a correlation, therefore a p-value > 0.05 was considered as indicating a statistically significant difference between the two sets. p = 0.23 (**). (TIF) [file ppat.1008209.s002.tif]

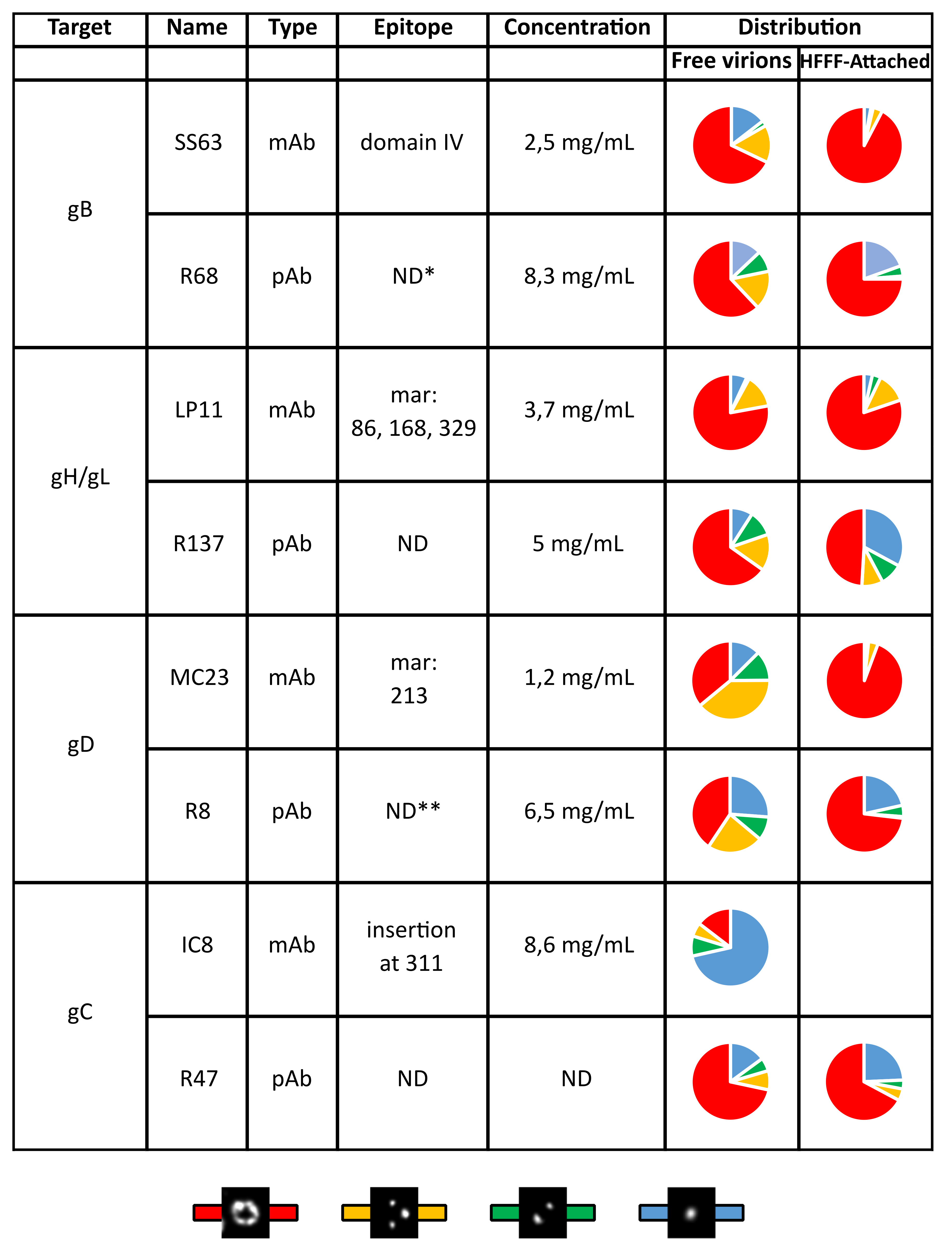

Supplement: S3 Fig — Color-coding is identical as that of Fig 2: red: rings; yellow: multiple spots; green: double spots and blue: single spots. “Epitopes” indicates the residues or domains involved in antibody binding. References are listed in the Methods section. mar: mAb resistant mutation. (*) partial blocking of domains I (20%), II (15%) and IV (40%) of gB. (**) blocks several known epitopes of gD (residues 10–20, 67, 246, 75–79, 213 (MC23) and 262–279). (TIF) [file ppat.1008209.s003.tif]
