# Supplementary material for: Dynamic organization of Herpesvirus glycoproteins on the viral envelope revealed by super-resolution microscopy
Source: PLoS Pathog. 2019 Dec 2;15(12):e1008209. doi: 10.1371/journal.ppat.1008209 (PMC6907858; doi:10.1371/journal.ppat.1008209)
Supplement: Attachment — Submitted filename: Answer to reviewers.docx [file ppat.1008209.s004.docx]

**Line numbers refer to the revised manuscript with no highlighted changes.**

**Reviewer #1**

**1.** *Have epitopes recognized by the different mAbs been identified, and if so can the authors provide their localization? This information could be helpful for interpretation of results (see lines 170-179, 231, and 347-348).*

All the information regarding epitopes recognized by the different antibodies used in this study has now been added to supplementary figure 3 and corresponding references were added in the Methods section (line 441).

**2.** *The authors report 4 different profiles of gp localization, but they mainly distinguish the “ring-like” as opposed to the other three profiles in their interpretation of results (Fig 1). First, do the authors suggest that the single, double and multiple profiles correspond to a similar functional state as opposed to the ring-like, or that each of these profiles correspond to different states/ functions? Second, were there non “ring-like” profiles containing more than 3 spots, or is 3 the largest distinguishable number of individualized spots in the multiple profile (i.e., how do the authors differentiate the multiple and “ring-like” profiles)? This point should be clarified since the whole discussion is based on this distinction : in particular, adding the “multiple” and the “ring-like” profiles would eliminate the distinction between the cell-free and the cell-attached localization of gD in HFF cells, and in HeLa cells analyzed with a pAb.*

This is an important point indeed that was indirectly raised in the discussion (lines 388-396 of 1^St^ version) but certainly deserves a clearer definition. We define a “ring” as a continuous structure where separated spots could not be observed. “Multiple spots” were arbitrarily defined as three or more spots on the model of what was done with HIV-1 (Chojnacki et al., 2012), although in this paper the authors included rings with multiples, probably because more than 3 multiple spots were never or very rarely seen. In our case, three or four spots were most commonly observed, whereas occurrences of six or seven spots could be observed but that was very rare (one is visible for gH/gL in Figure 6B, bottom line of LP11+R8), indicating that a ring is not necessarily made of 5 multiple spots or more but more likely represent a structural organization of gps of its own.

In this regard, we interpret spots (unique or multiple) as clusters of gps as opposed to rings which more likely represent a more even distribution of gps around the particle. Clustering of gps can have biological significance on infectivity (positively or negatively) as was demonstrated with HIV-1, and we believe that the fact that clustering concerns essentially the receptor-binding proteins gD and gC (IC8 epitope) is significant (see answer to point #6 of this reviewer). Hence the distinction between “rings” and others. We believe that since 3-4 spots were the most common occurrence of multiple spots, it represents a stable state of clustered proteins, whereas cases of 5+ spots being rare could indicate an intermediate state where glycoproteins are in the process of relocalizing as rings.

We highlighted the justification for the distinction between “spots” and “rings” in lines 101-103 and lines 336-340 of the revised manuscript.

**3.** *The results obtained using polyclonal and monoclonal antibodies are not strictly identical, mABs tending to provide more ring-like images on cell-bound particles than pAbs (see Fig 2 gB, gD). Are these differences significant and if so, how can they be explained? (see Q1).*

The percentage of ring-like images tends indeed to be slightly higher with mAbs than with pAbs in conditions where cells are present or rather there are slightly more single spots present on images with cells because of a low-level background linked to the cell membrane (hence a lower % of rings). This background is present essentially with pAbs. However, we have found that these differences are not significant (Pearson’s qui-squared test for correlation at p>0.05 for significant differences), although the R8/MC23 distribution on HFFF cells has a p-value close to the threshold for significance (p=0.04).

**4.** *A constriction of the diameter of rings of gB, gH-gL and gD is observed when comparing cell-free and cell-attached virions, ranging from 12 to 21% (Fig 3). There is also a difference in the diameter of gp rings on cell-free virions using pAbs compared with mAbs (Fig 3A, see gB and gH/gL, and Fig3B, see gB and gD): is it significant and how can it be explained?(see Q1).*

The difference the reviewer is referring to is indeed significant. Rings observed in STED with pAbs generally have a larger diameter and a thicker ring line than when observed with mAbs. An instance of this is visible on Figure 6B. One possible explanation is that pAbs recognizing several epitopes give an overall stronger signal than mAbs. This signal being stronger requires that the depletion laser of the STED be set at a higher value than for the imaging of mAbs rings to achieve similar resolution. We imaged all conditions (mAbs and pAbs alike) with a similar STED set-up which may have resulted in a lower resolution obtained with the pAbs, hence an apparent larger ring diameter.

This is now specified in lines 477-481 of the revised manuscript.

Of note, while answering this reviewer’s question, we realized that there was a small mistake done regarding the constriction of gB with mAb SS63 in the first experiments: it is stated as 12% whereas it should read 10% (from 270 to 243 nm). This is now corrected in line 193 of the revised version.

**5.** *Figure 5 : the interpretation that spots of gD and gC colocalize (lines 243-244, and 250-251) is not obvious (compare lanes x and xi).*

There is little doubt that gD and gC are physically close on the viral envelope: it explains why their dual-labelling is particularly inefficient (Figure 5 and lines 230-231 of 1^st^ version) and it is in agreement with cross-linking experiments published earlier (Handler et al., JVI 1996).

Instances of colocalization *per se* are rarer in STED when it comes to spots since the higher the resolution the less probable two spots will overlap, unless the molecules are actually really close together, at which point dual-labelling can be tricky (as it was here). This is why we specified at lines 243-244 that “spots of gC and gD are often observed as adjacent or colocazing (line i))”. In the case of gC and gD, colocalization is quite obvious in line (i) and much less in line (x) indeed. However, the IC8 epitope is clearly particular as described in lines 211-217 and is not representative of all gC molecules, as opposed to pAb R47. This is why we focused our conclusions on results as shown in line (i).

To avoid confusion, we rephrased the conclusion of this paragraph as follows : “Similarly, a fraction of gD molecules seem to be sufficiently close to gC to be observed as adjacent and sometimes colocalizing spots in our set-up.” (lines 252-253). In addition, we completed the sentence line 233 in the results by “possibly because the two molecules are very close together {Handler, 1996 #28}.”

**6.** *Figure 6 C : The results show that gD is clustered at the surface of cell-free particles, and redistributed in a ring-like organization in cell-bound particles. As the authors remind (p 14 line 316), glycoproteins involved in cell binding and fusion should regroup locally at the point of contact with cells : indeed maturation of HIV particles is contemporary with the egress of mature particles from cells, and env clustering is supposed to render the virus productive for infection (Chojnacki, Nat Commun 2017). Therefore, the results shown in this manuscript seem to contradict previous results obtained in another model. Can the authors clarify further this point in their discussion ?*

We agree that while comparing the two viruses, the opposing effects of clustering are not clear enough. We did not go into details in comparing the two viruses (although this would definitely be worth it) because the discussion is already lengthy and that we aimed at focusing on the discussion of the results described here on HSV1.

First, we think that the ring constriction observed for gB, gH/gL and gD could be associated to their regrouping at the point of contact between viral envelope and plasma membrane, as specified in the original manuscript (lines 306-310).

Our hypothesis concerning clustering of gD is that it allows its efficient inhibition in immature particles, as stated in lines 388-396 of the discussion (1^st^ version) and this is where differences with *env* start.

There are very few molecules of *env* at the surface of HIV-I and *env* is necessary for receptor binding and fusion. Clustering of fusion-related glycoproteins is generally viewed as important to optimize the energy-demanding process of membrane fusion, in particular in cases where the number of proteins is limited as for *env*. In the case of HSV-1, however, gD is necessary only for receptor binding and, although it is necessary to trigger the activation of the fusion-related proteins gH/gL and gB, it is not directly involved in membrane fusion. Moreover, there are around 300 molecules of gD at the surface of HSV1 particles (or 150 dimers, as opposed to 7-10 trimers of *env*)(Clarke et al., Biophys. J. 2007) and one is sufficient to allow entry (Clarke et al., JACS 2013). Thus, clustering is unlikely to be necessary for its function.

In conclusion, although the clustering of gD resembles that of *env*, it seems to have opposite functions, essentially because the two gps have differing functions and numbers on the viral envelope. Whereas clustered *env* is associated with mature HIV-1 particles, it is the non-clustered form of gD which is associated with mature HSV-1, or at least with the cell-bound particles. We believe that clustering of gD allows for its efficient inactivation on immature particles as detailed in the original manuscript (lines 388-396).

In order to clarify the difference between the two viruses, the following statements were added in the discussion: “Although the clustering of gD resembles that of env, it seems to have opposite functions. Clustered Env is associated with mature HIV-1 particles whereas it is the non-clustered form of gD which is associated with cell-bound HSV-1” (lines 389-391 of the revised manuscript).

**7.** *Since redistribution of gD was observed at 4°C, i.e. in conditions which permit attachment but preclude fusion, can the authors comment on glycoprotein mobility in membranes and in particular at that temperature?*

The point raised here by the reviewer anticipates on a question that should be more properly addressed in a follow-up study, i.e what is the molecular mechanism behind gD relocalization? Here, we observe an extreme form of relocalization: from clusters to even distribution round the particle.

As stated in the discussion (lines 294-296 of 1^st^ version), many Herpesviral glycoproteins are linked to the tegument through their cytoplasmic tail. This should hinder glycoprotein mobility and could be responsible for the clustering of gD. The relocalisation of gD is likely to occur through cleavage of some tegument proteins or conformational changes within the tegument which could either release gD from its tegument tether or relocalize it according to the tegument conformation.

It is unclear what triggers gD relocalization : particles maturation and/or viral pre-attachment at the surface of the cell (such as interaction of gC with heparan sulfates) (see lines 372-373 of 1^st^ version). In the first case, the temperature used during our binding assays should have no impact since the relocalisation would have taken place independently of virus binding. In the second case, it implies that no energy is required for the process and that mobility of gD on the viral envelope is sufficient for its even distribution over the course of 30 mins (the incubation time used on our assays). This could be plausible in the case where tethering of gD is the active process whereas its release is a passive, energy-free process.

However, these hypotheses remain to be experimentally tested since our data are not sufficient to answer this question. This could be done on the line of what was described with HIV-1 for instance.

**Reviewer #2**

**1.** *Upon binding to the cells, the authors claimed the rings of glycoproteins were constricted. However, it is many possibilities in addition to the change on the position of glycoproteins. The authors should mention the other possibilities, such as global change of the modifications of epitopes on the glycoproteins.*

We agree that the observed constrictions could have alternative explanations than those put forth in the discussion.

Conformational changes of glycoproteins (gps)(that could lead to “modifications” of the epitopes such as their position) are very unlikely to be the reason of the observed constriction of ring diameters for several reasons: (i) constriction is observed for all gps (except gC), this means that all gps must undergo conformational change after maturation and/or adsorption if the hypothesis is correct; (ii) even if conformational change of all gps occurs, then the constriction should be related to the size of the gps. gD, gH/gL and the pre-fusion form of gB have been shown or estimated to be 6 nm, 10 nm and 10 nm long respectively (Krummenacher et al., 2005; Chowdary et al., 2010 and Stannard et al., 1987). Thus, in the most extreme scenario whereby all gps change conformation reducing their size by half, a reduction of 10 nm (2x5nm) of ring diameters should be expected. This is less than the lowest constriction of rings we actually observed (20 nm for SS63 in experiment set #2) and much less than the average constriction observed (40 nm).

In addition, it should be kept in mind that ring constriction was also observed with pAbs, which can recognize several epitopes on each gps, which means that we are looking at a general conformational change and not at the movement of a single epitope within the protein.

We have added this discussion to the revised manuscript (lines 308-314).

**2.** *Can the authors estimate the number (ratio) of gD among virions?*

It has been previously demonstrated that the average number of gD molecules per virion is around 300 molecules (Clarke et al., Biophys J 2007). In addition, Handler et al. showed that gD is approximatively 11-14 times more abundant than gB (Handler, 1996). Importantly, a single molecule or two of gD is enough to engage receptors and trigger entry (Clarke et al., JACS 2013). This makes the “inhibition cluster” theory as described in lines 388-396 (original manuscript) more plausible since regrouping all gD molecules in inhibitory clusters should allow for its efficient inhibition.

**3.** *The authors should show the methods of collecting the virions. Such as cell lines, MOI or times after infection. These conditions effect composition of virions dramatically.*

The whole procedure is now detailed in the Material and Methods section (lines 423-436).

**Other corrections/modifications**

**1. Line 193:** Corrected a typo regarding the constriction of gB with mAb SS63 in the first experiments: it is stated as 12% whereas is should read 10% (from 270 to 243 nm).

**2.** **Line 4, 7-8**: Affiliation of first author Dr. Frauke Beilstein has been modified at her request.

**3. Line 506-507:** An additional sentence has been added to the acknowledgments.
